# Spatial and Temporal Variability of Macroinvertebrates in Spawning and Non-Spawning Habitats during a Salmon Run in Southeast Alaska

**DOI:** 10.1371/journal.pone.0039254

**Published:** 2012-06-20

**Authors:** Emily Y. Campbell, Richard W. Merritt, Kenneth W. Cummins, M. Eric Benbow

**Affiliations:** 1 Department of Entomology, Michigan State University, East Lansing, Michigan, United States of America; 2 Department of Fisheries Biology, Humboldt State University, Arcata, California, United States of America; 3 Department of Biology, University of Dayton, Dayton, Ohio, United States of America; Freie Universitaet Berlin, Germany

## Abstract

Spawning salmon create patches of disturbance through redd digging which can reduce macroinvertebrate abundance and biomass in spawning habitat. We asked whether displaced invertebrates use non-spawning habitats as refugia in streams. Our study explored how the spatial and temporal distribution of macroinvertebrates changed during a pink salmon (*Oncorhynchus gorbuscha*) spawning run and compared macroinvertebrates in spawning (riffle) and non-spawning (refugia) habitats in an Alaskan stream. Potential refugia included: pools, stream margins and the hyporheic zone, and we also sampled invertebrate drift. We predicted that macroinvertebrates would decline in riffles and increase in drift and refugia habitats during salmon spawning. We observed a reduction in the density, biomass and taxonomic richness of macroinvertebrates in riffles during spawning. There was no change in pool and margin invertebrate communities, except insect biomass declined in pools during the spawning period. Macroinvertebrate density was greater in the hyporheic zone and macroinvertebrate density and richness increased in the drift during spawning. We observed significant invertebrate declines within spawning habitat; however in non-spawning habitat, there were less pronounced changes in invertebrate density and richness. The results observed may be due to spawning-related disturbances, insect phenology, or other variables. We propose that certain in-stream habitats could be important for the persistence of macroinvertebrates during salmon spawning in a Southeast Alaskan stream.

## Introduction

The idea of the refuge is critically important and some argue that it should be considered an integrating concept in ecology and evolution [1]. This is because it encompasses a variety of phenomena including: enemy-free space, cover, crypsis, functional responses, factors of predator and prey behavior, competition for resources and shelter [1]. Past investigations of macroinvertebrate utilization of refugia in response to disturbance have largely focused on flow [2]–[4], while the use of refugia in response to salmon spawning is less well studied [5]. Macroinvertebrate distributional changes during salmon spawning could have important implications for the management and conservation of benthic macroinvertebrates and the organisms that consume them, both within streams and the adjacent riparian forests.

As anadromous and semelparous organisms, Pacific salmon (*Oncorhynchus* spp.) offer annual nutrient pulses to streams when they return to spawn and these subsidies can have ecological effects on lotic organisms over time [6], [7]. Salmon resource subsidies have been documented to positively influence benthic macroinvertebrates [5], [8], perhaps by the provision of nutrients and carbon that have been shown to increase during salmon runs [9]. Stream environmental factors such as sediment size and large wood recruitment [9]–[11] along with spawning disturbance intensity [5], [12] can alter the degree of marine-derived nutrient transfer available to benthic communities.

In strong contrast to the nutrient enrichment effect, adult salmon disturb benthic communities in spawning habitat by redistributing substrata during redd (nest) construction [5], [14]. Spawning disturbances can change the distribution, abundance and community composition of macroinvertebrates, causing substantial reductions in riffles where the redds are built [15]–[18]. Salmon redd construction can cause disturbances locally by displacing macroinvertebrates into drift at the excavation site due to suction and frictional forces; and also can disturb invertebrates downstream of the excavation site as displaced fine sediments can abrade and fill in interstitial spaces [5].

Habitat heterogeneity in streams is important as it sustains high biological diversity [13], [19], [20]. For example, riffles are often dominated by scrapers, such as heptageniid mayflies, that feed on benthic biofilm and collector filterers, such as simuliid dipterans, that collect fine particulate organic matter from stream drift. Pools however, typically sustain more shredders, such as limnephilid case building caddisflies, and collector gatherers, such as some genera of Chironomidae [21]. Heterogeneity in streams can also offer organisms refugia, which we define here as distinct non-spawning habitats that are not normally disturbed [22], [23].

Areas that are less likely to be influenced by spawners include: pools or other slack water habitats unusable for salmon eggs [7], stream margins which are too shallow for redd construction [2], and the hyporheic zone which is too deep to be disturbed [5], [7]. Macroinvertebrates could use such habitats as refugia, avoiding the effects of bioturbation [14]. Organisms may inhabit refugia temporarily during disturbances and then disperse once it has passed [22], [24], [25], or they may stay within refugia after disturbances.

We investigated the spatial and temporal variability of macroinvertebrates in spawning and non-spawning habitats in a stream where salmon were spawning. We hypothesized that during spawning there would be: i) a reduction in the density, biomass and taxonomic richness of macroinvertebrates in riffles; ii) increased macroinvertebrate density, biomass and taxonomic richness in pools, margins and the hyporheic zone; and iii) increased daytime macroinvertebrate drift.

## Results

The estimated density of adult salmon in our 300 m reach was 0.88 m^−2^ during this study (2008), compared to 0.51 m^−2^ in 2007 and 0.36 m^−2^ in 2006 [10], [11]. Chlorophyll *a* was not statistically tested, but we observed a decline in pools and an increase in riffles and margins during the salmon run (Table 1).

**Table 1:** Characteristics of riffles, margins and pools in Twelve Mile Creek, Alaska.

	Avg. Area (m^2^)	Avg. Temp. (°C)	% Dissolved Oxygen	pH	Mean Sediment Size(mm)	% Canopy Cover	Mean Chlorophyll *a*(mg m^−2^) Before Salmon	Mean Chlorophyll *a*(mg m^−2^) During Salmon
Riffle	185.2 (0.25)	9.7 (0.09)	99.2 (0.54)	8.1 (0.02)	30.9 (3.03)	19 (5.79)	8.4 (1.22)	10.3 (1.15)
Margin	N/A	10.8 (0.54)	97.9 (0.85)	8.1 (0.06)	21.9 (5.1)	26 (7.04)	10.1 (1.8)	10.2 (1.6)
Pool	35.5 (0.23)	9.8 (0.16)	85.3 (9.7)	7.8 (0.2)	23.8 (3.49)	69 (12.39)	14.9 (1.78)	10.3 (1.08)

Means are presented and numbers in parenthesis represent standard errors.

### Macroinvertebrates in Riffles

**Figure 1:**
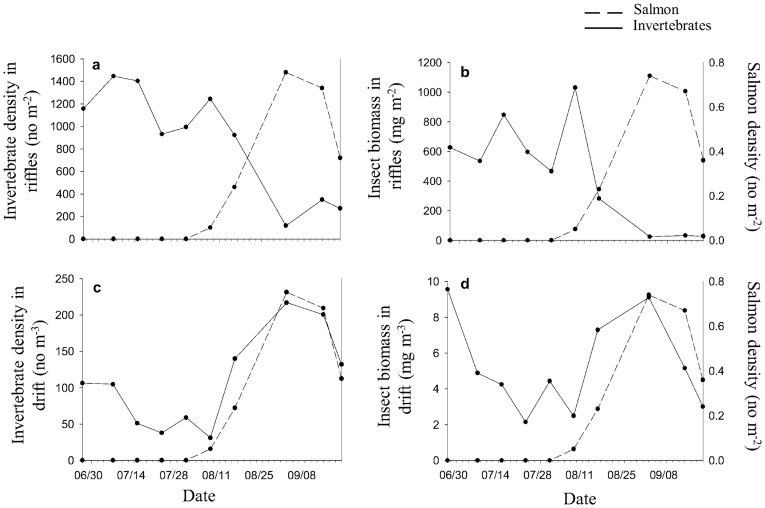
Invertebrates in riffles and drift throughout the salmon run. Invertebrate density (a) and insect biomass (b) in riffles, and invertebrate density (c) and insect biomass (d) in drift plotted against salmon density (right-hand axis). Note the differences in scale on the y-axis in all figures.

**Figure 2:**
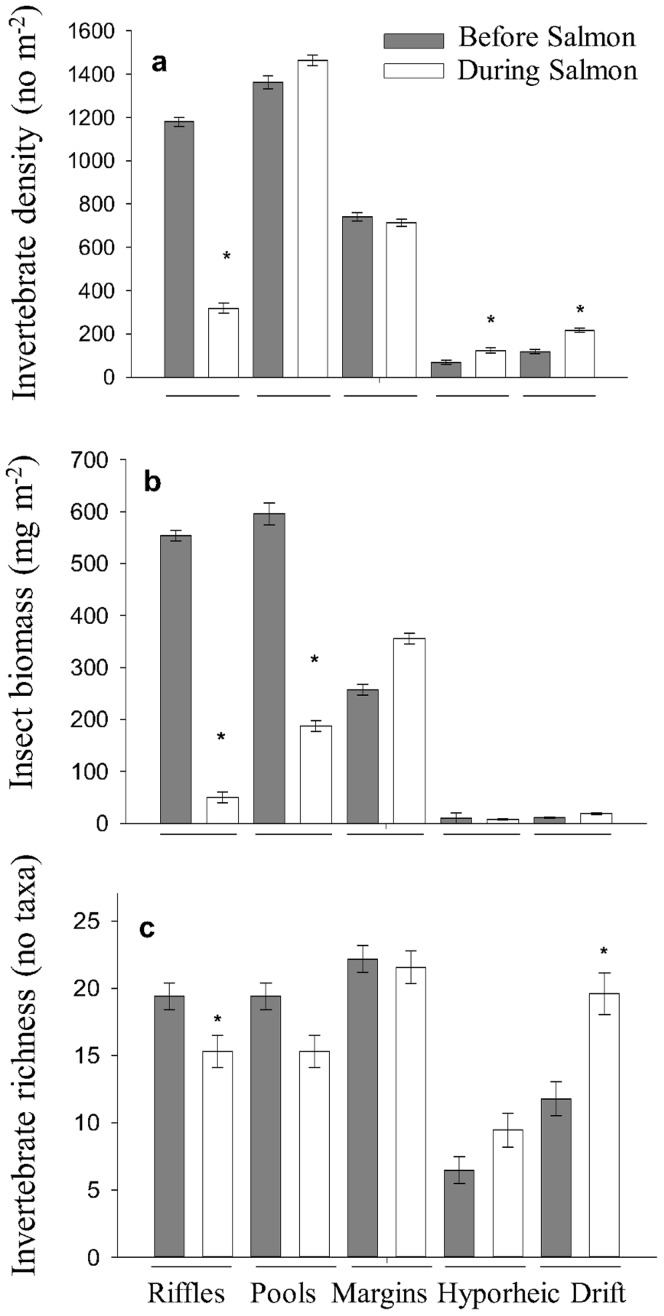
Invertebrates in all habitats before and during spawning. Invertebrate density (a), insect biomass (b) and taxonomic richness (c) before (dark grey bars) and during (white bars) the salmon run in riffles, pools, margins, the hyporheic zone, and drift. Graphs shows +/−1 s.e. and * means *p*<0.05.

Macroinvertebrate assemblages in riffles significantly changed during salmon spawning. Regression analysis showed that invertebrate density was negatively correlated with that of salmon density (*R^2^* = 0.84, *p*<0.001). A sharp decline was observed in macroinvertebrate density and insect biomass in riffles upon the arrival of salmon spawners (Figs. 1a, 1b). Repeated measures analysis of variance (rmANOVA) showed that macroinvertebrate density (*p*<0.001, Fig. 2a), insect biomass (*p*<0.001, Fig. 2b) and taxonomic richness (*p* = 0.009, Fig. 2c) declined in riffles during spawning.

**Figure 3:**
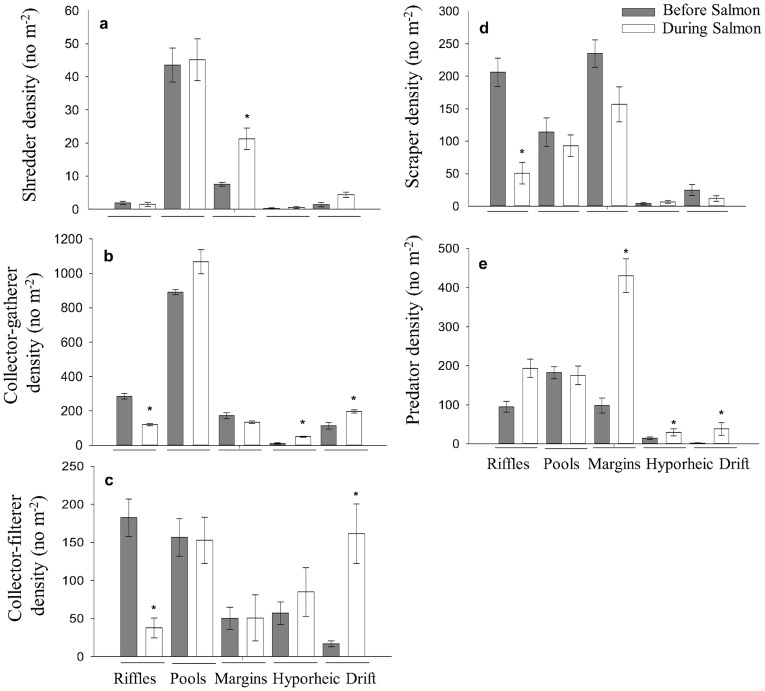
Functional feeding group differences before and during spawning. Shredder (a), Collector-gatherer (b), Collector-filterer (c), Scraper (d), and Predator (e) densities before (dark grey bars) and during (white bars) the salmon run in riffles, pools, margins, the hyporheic zone, and drift. Graphs shows +/−1 s.e. and * means *p*<0.05.

**Figure 4:**
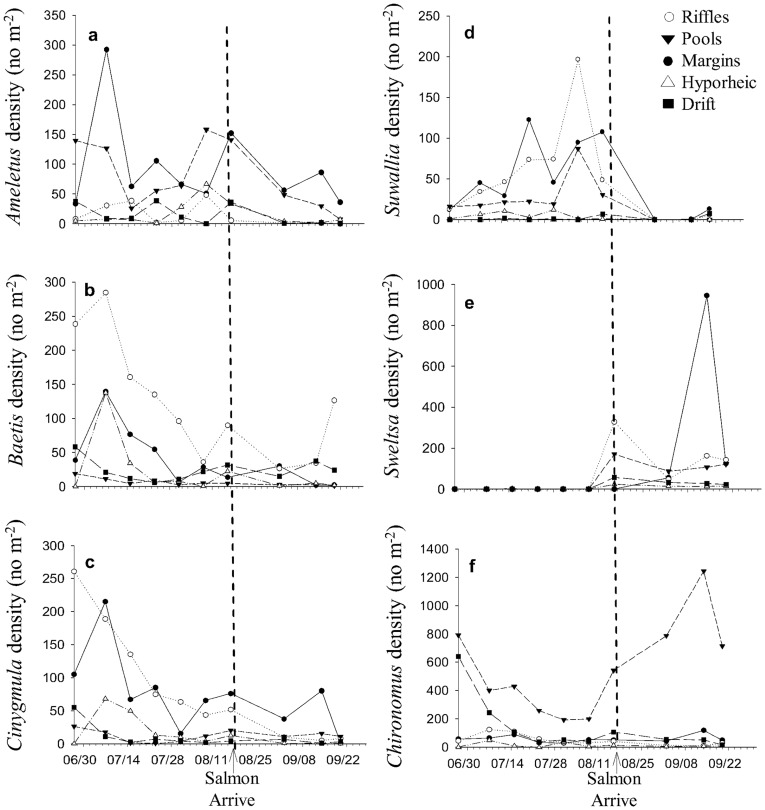
Genus-level differences throughout the salmon run. Densities of the six dominant taxa: *Ameletus* (a), *Baetis* (b), *Cinygmula* (c), *Suwallia* (d) *Sweltsa* (e) and *Chironomus* (f) throughout the salmon run in riffles, pools, margins, the hyporheic zone and drift.

We observed a change in functional feeding group structure in riffles during spawning. The density of collector-gatherers (*p*<0.001, Fig. 3b), collector-filterers (*p*<0.001, Fig. 3c) and scrapers (*p*<0.001, Fig. 3d) declined in riffles during spawning. Genus-specific changes were also observed during spawning in riffle habitats (Fig. 4a–f). The dominant taxa were: *Chironomus* (19.9% of total taxa), *Sweltsa* (7.6%), *Ameletus* (5.6%), *Baetis* (5.3%), *Cinygmula* (5.1%) and *Suwallia* (3.2%). *Sweltsa* density (*p*<0.001) was the only genus that increased in riffles during the salmon run (Figs. 4e). *Ameletus* (*p* = 0.019), *Baetis* (*p* = 0.038), *Cinygmula* (*p*<0.001) and *Suwallia* (*p*<0.001) densities all declined, whereas *Chironomus* density did not change in riffles during spawning (Figs. 4a–f).

### Macroinvertebrates in Drift

Drift density was positively correlated with salmon density (*R^2^* = 0.61, *p*<0.01). The density (*p* = 0.049, Fig. 1c, Fig. 2a) and taxonomic richness (*p* = 0.001; Fig. 2c) of macroinvertebrates increased in stream drift during salmon spawning. The densities of collector-gatherers (*p* = 0.05, Fig. 3b), collector-filterers (*p* = 0.02, Fig. 3c) and predators (*p*<0.001, Fig. 3e) increased in stream drift during spawning. *Sweltsa* density (*p*<0.001, Fig. 4e) increased, while all other dominant genera did not significantly change in stream drift during spawning.

### Community Structure

**Figure 5:**
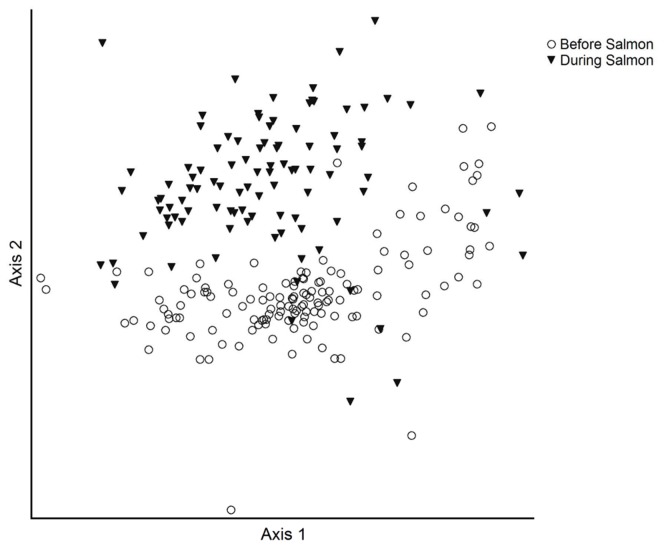
Macroinvertebrate communities before and during spawning. Non-Metric Multi-Dimensional Scaling ordination showing the separation of invertebrate community structure before and during the salmon run. A total of 65% of the variation in macroinvertebrate community structure was explained by: 1^st^ axis  = 36% and 2^nd^  = 29%.

The NMDS ordination and MRPP revealed a significant (*p*<0.001) difference in macroinvertebrate community structure before and during the run (Fig. 5). A total of 83% of the variation in macroinvertebrate community structure was explained by a three axes solution: 1^st^ axis  = 36%, 2^nd^  = 29% and 3^rd^  = 18% and the mean stress was 15.3. We ran separate ordinations for the invertebrate assemblages in all habitat types before and during spawning, and did not find a significant difference. We therefore show the more robust ordination comparing overall before and during salmon invertebrate communities. The dominant indicator taxa in each habitat type before and during the salmon run are listed in Table 2.

**Table 2:** Dominant indicator taxa based on indicator species analysis (ISA) pooling before and during the salmon run in each habitat type and drift.

	3 Most Significant Taxa	Indicator Values
Riffle	*Planaria*, *Epeorus*, *Baetis*	40%, 43%, 37%
Margin	*Ameletus*, *Cinygmula*, *Suwallia*	40%, 35%, 28%
Pool	Oligochaete, *Probezzia*, *Oreodytes*	61%, 53%, 50%
Hyporheic	Ostracoda, Copepoda, *Paraleptophlebia*	47%, 36%, 22%
Drift	Chironomidae, Acari, Araneae	84%, 54%, 42%

### Macroinvertebrates in Refugia

Macroinvertebrate density in margins, pools and the hyporheic zone were not correlated to salmon density. Within stream margins, macroinvertebrate density, biomass and taxonomic richness did not change (Figs. 2a–c). In pool habitats, the biomass of insects declined (*p* = 0.002, Fig. 2b), while invertebrate density (Fig. 2a) and richness (Fig. 2c) did not change during spawning. The hyporheic zone showed an increase in invertebrate density (*p* = 0.019, Fig. 2a) but no change in insect biomass (Fig. 2b) or taxonomic richness (Fig. 2c) during the salmon run.

Functional feeding group structure changed in non-spawning habitats during the salmon run. In stream margins, there was an increase in shredder (*p* = 0.02, Fig. 3a) and predator (*p*<0.001, Fig. 3e) densities during the salmon run. In the hyporheic zone, collector-gatherer (*p* = 0.007, Fig. 3b) and predator (*p* = 0.02, Fig. 3e) densities increased. In stream margins, *Baetis* (*p*<0.001), *Cinygmula* (*p* = 0.012) and *Suwallia* (*p*<0.001) densities declined, while *Sweltsa* (*p*<0.001) density increased during the salmon run (Figs. 4b–e). In pools, the density of *Suwallia* (*p*<0.001) declined and the densities of *Sweltsa* (*p*<0.001) and *Chironomus* (*p*<0.001) increased (Figs. 4d–f). In the hyporheic zone, *Suwallia* (*p* = 0.004) density declined and the density of *Sweltsa* (*p*<0.001) increased during the run (Figs. 4d–e).

## Discussion

In this study, we quantified macroinvertebrate distributional changes in spawning (riffle) and non-spawning (pool, margin, hyporheic) habitats during a pink salmon (*Oncorhynchus gorbuscha*) spawning run. Consistent with the results of previous studies, macroinvertebrate density, biomass and taxonomic richness declined in riffles during the salmon run [5], [11], [13], [26]–[28]. We speculate that the precipitous decline in riffle macroinvertebrate density and biomass was due to spawning-related activities. This explanation is bolstered by a controlled field experiment where we installed mesh exclosures in spawning habitat that prevented salmon disturbance, which were compared to areas where salmon had access. We found a greater abundance of macroinvertebrates in exclosure plots relative to control plots during spawning, suggesting that salmon were the causal mechanism for the observed invertebrate decline within spawning habitat [29].

We observed an increase in macroinvertebrate drift density, biomass and taxonomic richness during spawning as compared to pre-salmon related drift. This effect has been demonstrated in several other studies [5], [15], [30]. The sharp increase in drift density and biomass that occurred shortly after salmon arrival in this study is likely due to salmon nest construction which displaced macroinvertebrates into drift at the excavation site due to suction and frictional forces [5]. There are other possible explanations however, for example, macroinvertebrates in Alaska may have prolonged drift periods in the summertime due to the long photoperiod causing higher drift densities. Additionally, the flood that occurred on 23 August could have accounted for the observed increase in invertebrate drift.

In comparison to riffle habitats, the macroinvertebrate communities in pool, margin and hyporheic habitats remained relatively stable throughout the salmon run. Invertebrate density and richness did not change in stream margins and pools, but insect biomass declined in pools during the salmon run. We propose that this was due to an emergence event. Insect emergence is high in the late summertime in Alaska and in high latitude streams in general, so it is possible that natural emergence could explain the reduction of insect biomass in pools during the run [30]. Overall and taxa-specific invertebrate density increased in the hyporheic zone during spawning, these results may be explained by invertebrates avoiding the physical disturbances from salmon spawners, but could also be due to insect life history cycles or food limitations in disturbed riffles causing invertebrates to vertically migrate into the hyporheic zone to feed.

Disturbance is a central organizing factor in stream communities [31] and is fundamental to the concept of patch dynamics, whereby the temporal and spatial variability of ecosystems are established by disturbance impacts [32]. We demonstrate that invertebrate assemblages undergo spatial shifts within the stream channel during spawning and suggest that adult salmon are the cause of the observed invertebrate reductions in patches (riffles) where spawning activities are greatest [15], [17]. Managers can use these data as incentive to maintain channel complexity which may be an important factor regulating invertebrate persistence during disturbances [11]. The concept of a refuge is important in both basic and applied ecology, particularly as a stabilizing force during natural and anthropogenic disturbances [1]. Certain in-stream habitats may offer refuge to macroinvertebrates and be a fundamental determinant of macroinvertebrate resilience to bioturbation from spawning salmon in Southeast Alaska.

## Materials and Methods

### Study Sites

This study was conducted within a 300 m reach of Twelve Mile Creek (N55°482, W132°631) on Prince of Wales Island within the Tongass National Forest, Southeast Alaska, USA (Table 1). Catchments on Prince of Wales Island are composed of coniferous temperate rainforest that have been managed primarily for timber harvest. Much (68%) of the Twelve Mile Creek catchment has been harvested for timber, mostly in the 1960’s. Dominant riparian tree species along the stream include red alder (*Alnus rubra* (Bong)), western hemlock (*Tsuga heterophylla* (Rafinesque)) and Sitka spruce (*Picea sitchensis* (Bongard)).

### Macroinvertebrate Sampling and Processing

Macroinvertebrate samples were collected as five replicates each from riffles, pools, stream margins and hyporheic wells every 10 days from 27 June until 20 September 2008. The 10 sampling dates included five before the salmon arrived and five during their spawning. A flood prevented sampling on 23 August.

For riffles, pools and margins, benthic macroinvertebrates were collected using a large PVC pipe sampler (diameter, 36 cm; area, 0.4 m^2^). The pipe was pushed down into the sediments (approx. 10–14 cm) to minimize water exchange. Samples were collected by disturbing the substratum within the pipe to a depth of about 10 cm for 30 s. A 250 µm mesh net was then used to filter out organic matter and invertebrates and the material collected was preserved in 70% ethanol. A stream ‘margin’ was defined as the periphery of the channel with a width approximately equal to 10% the total stream width (mean channel width 13.05 m). ‘Pools’ were defined as low-velocity (0–0.02 m/s) areas caused by large wood debris or boulders, that were connected to the main channel at base flow, were not too deep (<0.65 m) and were adjacent to spawning habitats.

Hyporheic macroinvertebrates were collected using 0.6 m long PVC hyporheic wells installed 30 cm into the sediment at random within each riffle (1 well per riffle, 5 wells total). All wells were capped to avoid surface and water column invertebrates from intruding. The bottom 0.15 m of the wells had 30 holes (8 mm diameter) drilled into the sides to allow invertebrates to be withdrawn from a larger volume of water surrounding the bottom of the wells [33]. A bilge pump was used to pump 2 L of hyporheic water per well, which was then filtered through a 250 µm sieve and preserved in 70% ethanol.

Three drift nets (mesh, 250 µm) evenly spaced across the channel and 3 cm above the bed [33] were used to collect macroinvertebrate drift for 30 min. Mean current velocity (at beginning and end of 30 min) and water depth were measured and the volume of water filtered was estimated. Drifting material was preserved in 70% ethanol. As behavioral drift is greatest at dawn and dusk [34], drift was taken at noon to collect organisms principally dislodged due to spawning activities. Insects were identified to species or genus, non-insects to family or order and functional group designation based on Merritt et al. [21]. The lengths (nearest 0.5 mm) of insects were measured to allow estimation of biomass based on INVERTCALC, using length-weight regressions [35].

### Salmon

Summer runs in Twelve Mile Creek were dominated by pink (*Oncorhynchus gorbuscha* (Walbaum)) salmon, with chum salmon (*O. keta* (Walbaum)) being the next dominate salmon species present during the study. Resident juvenile coho salmon (*O. kisutch* (Walbaum)), Dolly Varden char (*Salvelinus malma* (Walbaum)) and sculpin (*Cottus* spp.) were also present. All live pink and chum salmon were quantified in 4 meter wide belt transects perpendicular to stream flow every 10 m for the entire 300 m reach. These counts were then scaled up to estimate the total number of salmon present in the 300 m stream reach on each date. Salmon were counted approximately twice weekly from the start of the spawning run on 5 August until only carcasses remained in the stream. Live salmon density increased from the start of the run throughout most of the summer and began to decline about 2 weeks before our last sampling date on 24 September 2008.

### Habitat Characteristics

In each habitat, we measured pH, % dissolved oxygen and specific conductivity using a Hydrolab MS 5 Mini Sonde (HACH Environmental, Loveland, Colarado, USA). Habitat area was measured with an electronic distance measurer (EDM), % canopy cover using a spherical densiometer, sediment size using a Wentworth scale gravelometer and water velocity and depth using a digital flow meter [33], each three times throughout the study. Additionally, we estimated benthic algal biomass (as chlorophyll *a*) by randomly selecting five cobbles which were immediately transported in a cooler to the laboratory and processed within 6 hours. The cobbles were scrubbed across their entire surface onto pre-ashed glass filters (0.7 µm) type A/E (Pall Corporation, Ann Arbor, Michigan, USA) and then analyzed with a Trilogy Turner Design Fluorometer (Turner Designs, Inc., Sunnyvale, California, USA). Surface area of the cobbles was estimated by measuring length, width and depth and assuming an ellipse. The chlorophyll *a* per unit area was then pooled across the five cobbles to obtain a mean.

No specific permits were required for the described field of studies. The study location is not privately-owned or protected in any way and the field studies did not involve endangered or protected species.

### Statistical Analysis

Linear regression was performed to analyze whether salmon density influenced macroinvertebrate density in the different habitat types and in the drift. The density and biomass of invertebrates in riffles and in the drift were plotted over the course of the salmon run to determine how invertebrates responded to salmon in different habitats and the drift. The densities of the six dominant genera: *Chironomus* (Diptera: Chironomidae), *Sweltsa* (Plecoptera: Chloroperlidae), *Ameletus* (Ephemeroptera: Ameletidae), *Baetis* (Ephemeroptera: Baetidae), *Cinygmula* (Ephemeroptera: Heptageniidae) and *Suwallia* (Plecoptera: Chloroperlidae), were also plotted over the course of the sampling period to determine how specific taxa responded to spawning salmon.

Repeated measures analysis of variance (rmANOVA) was performed with a Bonferroni correction to determine whether salmon presence (before and during) altered macroinvertebrate abundance and taxonomic composition. The two main effects were presence of salmon and habitat, where salmon was treated as the repeated factor and the replicates of each habitat were treated as random effects. A compound symmetric covariance structure was specified using SAS (Version 11; SAS Institute, Cary, North Carolina, USA). Macroinvertebrate response variables were total density, total biomass, taxonomic richness, the densities of shredders, collector-gatherers, collector-filterers, scrapers and predators, and the densities of the six dominant genera. Violations of the assumptions of ANOVA were corrected by transforming the data (logarithmic or exponential, as appropriate). Results were considered significant when α <0.05.

A Non-Metric Multi-Dimensional Scaling (NMDS) ordination was carried out to evaluate differences in macroinvertebrate community structure among the different habitats, and in the drift, before and during the salmon run [36] using PC ORD (version 5; MJM software, Gleneden Beach, Oregon, USA). We ran a total of 250 iterations for the real data with a random seed start. A multiple response permutation procedure (MRPP), using Sørensen distances, was performed to test for significant differences in community structure among habitat types before and during the salmon run. When significant differences were found in macroinvertebrate community structure, Indicator Species Analysis (ISA) was used to determine which taxa were significant indicators of the communities in the different habitat types. Taxa were considered significant in the ISA when indicator values (% of perfect indication) were >55% with *p*<0.001. All aquatic insect taxa that represented >3% of all samples were used in the ordination procedures.
